# Detection of Broken Strands of Transmission Line Conductors Using Fiber Bragg Grating Sensors

**DOI:** 10.3390/s18072397

**Published:** 2018-07-23

**Authors:** Long Zhao, Xinbo Huang, Jianyuan Jia, Yongcan Zhu, Wen Cao

**Affiliations:** 1 School of Electro-Mechanical Engineering, Xidian University, Xi’an 710070, China; zhaolong@xpu.edu.cn (L.Z.); jyjia@xidian.edu.cn (J.J.); 2 School of Electronics and Information, Xi’an Polytechnic University, Xi’an 710048, China; zhuyongcan@xpu.edu.cn (Y.Z.); caowen@xpu.edu.cn (W.C.)

**Keywords:** conductor, broken strands, modal identification, acceleration, natural frequency

## Abstract

Transmission lines are affected by Aeolian vibration, which causes strands to break and eventually causes an entire line to break. In this paper, a method for monitoring strand breaking based on modal identification is proposed. First, the natural frequency variation of a conductor caused by strand breakage is analyzed, and a modal experiment of the LGJ-95/15 conductor is conducted. The measurement results show that the natural frequencies of the conductor decrease with an increasing number of broken strands. Next, a monitoring system incorporating a fiber Bragg grating (FBG)-based accelerometer is designed in detail. The FBG sensor is mounted on the conductor to measure the vibration signal. A wind speed sensor is used to measure the wind speed signal and is installed on the tower. An analyzer is also installed on the tower to calculate the natural frequencies, and the data are sent to the monitoring center via 3G. Finally, a monitoring system is tested on a 110 kV experimental transmission line, and the short-time Fourier transform (STFT) method and stochastic subspace identification (SSI) method are used to identify the natural frequencies of the conductor vibration. The experimental results show that SSI analysis provides a higher precision than does STFT and can extract the natural frequency under various wind speeds as an effective basis for discriminating between broken strands.

## 1. Introduction

Aeolian vibrations often occur on transmission lines when a steady wind blows toward the conductors. Long-term vibration often causes a transmission line to break at the point of clamped contact [1,2]. To avoid such an accident, the vibration of transmission lines must be monitored. The existing method for such monitoring calculates the dynamic bending strain value by measuring the vibration amplitude of a conductor at a distance of 89 mm from the last contact point between the conductor and clamp; the method then calculates the fatigue life based on the dynamic bending strain and number of vibration cycles. Cantilever beam sensors [3,4], radar sensors [4,5], and acceleration sensors [6] have been used to implement this monitoring technique. Some of these sensors can accurately measure amplitude and frequency; however, they cannot accurately predict fatigue life. According to Reference [7], vibration will cause wear between strands; this wear is another factor that reduces operating life, in addition to metal fatigue effects, and leads to the need for frequent manual inspections to avoid broken line accidents.

In fact, strands can be repaired immediately after breaking, thereby preventing the line from breaking. A superconductor quantum interference device based on a high-temperature superconductor is used to monitor single line fracture in transmission lines. However, in this method, current must be injected into the running conductor, and the current and weight must then be detected [8]. The electromagnetic induction method is used to detect defects in transmission lines, and the defect voltage is determined by collecting the coil voltage [9]. Similarly to the magnetic induction method, some scholars have attempted to use eddy current sensors to measure the local defects in wires and identify the damage [10,11] to the wire through changes in the magnetic field reflected by the eddy current. However, the load current of transmission lines is variable, as is the magnetic field around them; these variations have a considerable influence on the magnetic induction or eddy current method. Moreover, these sensors must be installed above the broken strand position, and a broken wire may cause the sensor to loosen. A more effective noncontact measurement method is to identify a strand through image recognition. Image recognition can identify a broken strand by extracting the contour of the wire or comparing the chromatic aberration of the crack position [12,13]. However, there are still some problems with this method, such as the camera installation location, blind areas, and fouling caused by the surface of the wire.

For transmission lines, the most commonly used aluminum conductor steel reinforced (ACSR) cable is composed of several aluminum strands and steel cores. When one strand is broken, the bending stiffness decreases, which leads to changes in modal parameters. This technology, called modal identification, is applied to the structural monitoring of bridges [14,15] and wind turbines [16]. The mode of a structure can be identified through changes in the natural frequency, enabling the location of a fault or the length of a crack to be detected.

In this paper, a broken strand detection method using modal identification is proposed. This method is tested on an LGJ-15/95 transmission line. Experimental results show that the natural frequencies of each mode decrease after the strands break. Moreover, a fiber Bragg grating (FBG)-based monitoring system composed of an FBG-based acceleration sensor, wind speed sensor, analyzer, and monitoring center is designed. The system measures the vibration acceleration of a conductor and wind speed and then calculates the natural frequencies of the conductor. Finally, the monitoring system is tested on a 105 m transmission line span at Xi’an Polytechnic University, and the short time Fourier transform (STFT) method and the stochastic subspace identification (SSI) method are used to identify the natural frequencies of vibration of the conductor. The experimental results show that SSI analysis providers a higher precision than does STFT and can extract the natural frequency under various wind speeds. 

## 2. Broken Strands and Natural Frequencies

### 2.1. Principle

An overhead transmission line suspended between two towers has a certain sag caused by the combined effect of tension and gravity. The vibration mode of the wire is usually a sinusoidal wave, similar to the vibration of a string. The natural frequency for the vibration of a string can be calculated by the following equation:

(1) $$\omega_{n}=\frac{n\cdot \pi }{l}\sqrt{\frac{T}{m}}$$

where *ω* is the natural frequency, *l* is the length of the conductor, *T* is the tension of the conductor, *m* is the conductor mass per unit length, and *n* is the nth mode. 

**Figure 1:**
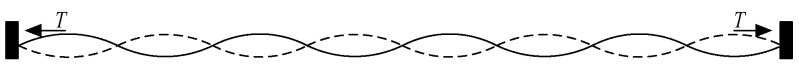
Model of the transverse vibration of a transmission line.

Equation (1) calculates the approximate value of the natural frequency of the transmission line but ignores the effect of stiffness on the natural frequency. In fact, the wire is more similar to a beam fixed at both ends and subjected to tension. A model of the wire’s transverse vibration is shown in Figure 1, and its natural frequency can be calculated by Equation (2):

(2) $$\omega_{n}={\left(\frac{n\cdot \pi }{l}\right)}^{2}\sqrt{\frac{EI}{m}}\sqrt{1+\frac{T\cdot l^{2}}{EI\cdot n^{2}\cdot \pi^{2}}}\;$$

where *EI* is the stiffness of the wire. Because of the unique properties of a given wire structure, the stiffness cannot be accurately calculated. However, the approximate stiffness of the transmission line can be obtained through the share-based calculation:

(3) $$EI=\frac{\pi }{64}\sum_{i=1}^{n}E_{i}d_{i}^{4}\;$$

where *E_i_* is the elastic modulus of the strands, *d* is the diameter of the strand, and *n* is the total number of strands.

**Figure 2:**
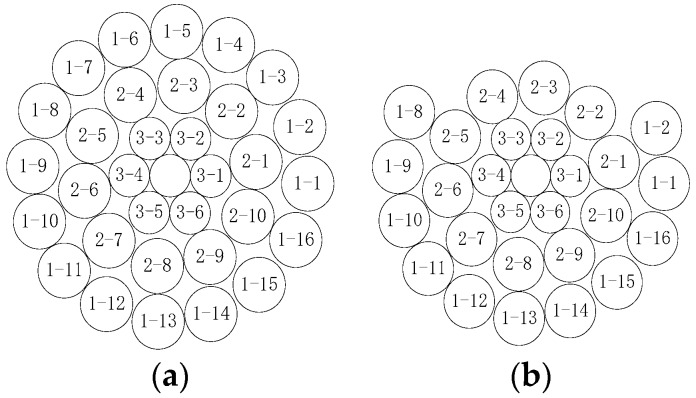
Cross-sectional view of a transmission line: (**a**) Before strands are broken; (**b**) After strands are broken.

Figure 2 shows a cross-sectional view of a transmission line. According to Figure 2, as well as Equations (2) and (3), when the strands break, the stiffness of the transmission line will decrease, and the natural frequency will decrease as the rigidity decreases. 

In addition, due to the change of temperature, the length and the stiffness of the conductor will change. The length of the conductor as a function of temperature can be expressed as:

(4) $$l_{T}=l_{0}\cdot (1+\alpha T)\;$$

where *l_T_* is the length of the conductor when the temperature is *T* °C, *l*_0_ is the length of the conductor when the temperature is 0 °C, *α* is the coefficient of linear expansion (18.9 × 10^−6^/°C for LGJ-95/15).

The stiffness of the wire as a function of temperature can be expressed as:

(5) $$EI=\frac{\pi }{64}\sum_{i=1}^{n}E_{i}\cdot (1-\eta T)\cdot d^{4}\;$$

where *η* is the temperature coefficient of the modulus of elasticity (4.72 × 10^−4^/°C for LGJ-95/15).

### 2.2. Experimental Setup

A vibration experiment was conducted to prove that modal identification can be used to identify broken strands.

**Figure 3:**
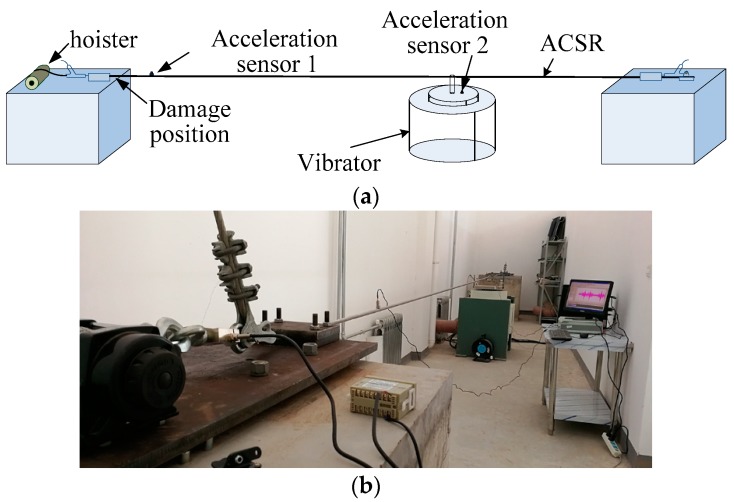
Experiment platform: (**a**) Schematic of the experimental platform; (**b**) Photograph of the experimental platform. ACSR = aluminum conductor steel reinforced.

Figure 3a shows a schematic of the experimental platform. The two ends of the conductor were fixed, and the initial tension was provided by the hoister. The tension sensor measured the tension value, and the two ends of the conductor were pressed by the pressure plate. For a conductor length of approximately 10 m, an LGJ-95/15 ACSR was used. In order to minimize the measurement error caused by temperature change, the indoor temperature was kept at 24 °C, with an error of not more than 1 degree Celsius. This type of ACSR had two layers of aluminum strands and two layers of steel cores. A vibrator was installed two-thirds along the length of the conductor, and the table of the vibrator was connected to the conductor.

**Figure 4:**
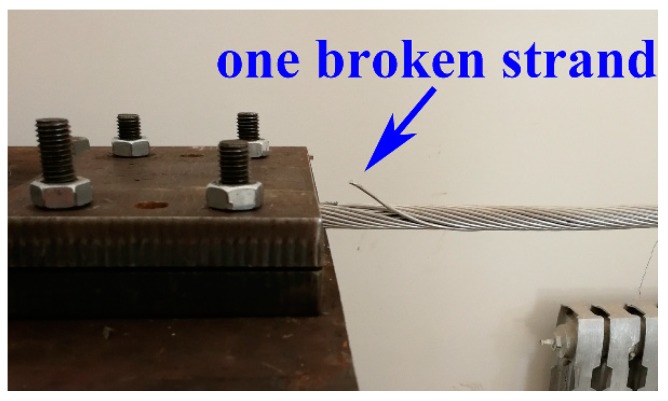
Broken strand location.

According to Equation (2), changes in the stiffness, length, tension, and mass per unit length of the conductor will cause changes in the natural frequency. To ensure that the natural frequency of the experimental wire was close to the natural frequency of the field wire, according to Equation (3), the conductor through the hoister exert tension was approximately 1200 N. One acceleration sensor was located 500 mm from an end of the conductor, while the other acceleration sensor was located at the table of the vibrator. The sampling frequency was set to 1 kHz. Although the frequency range of Aeolian vibration is theoretically 5–120 Hz, actual statistics show that the vibration frequency is less than 100 Hz. Therefore, the vibrator began to generate sinusoidal vibrations in a swept frequency mode with an amplitude of 0.5 g over a frequency range of 5–100 Hz. To ensure the accuracy of the measurement, each group was swept 2 times, and the experiment was repeated 4 times. Subsequently, the outer layer was cut off, and the sweep experiment was carried out again. For overhead transmission lines, strand breaking usually occurs at the last point of conductor contact with the clamp (the fixed end of the conductor). Thus, in this experiment, we cut the strand at the fixed end position, as can be seen in Figure 4.

**Figure 5:**
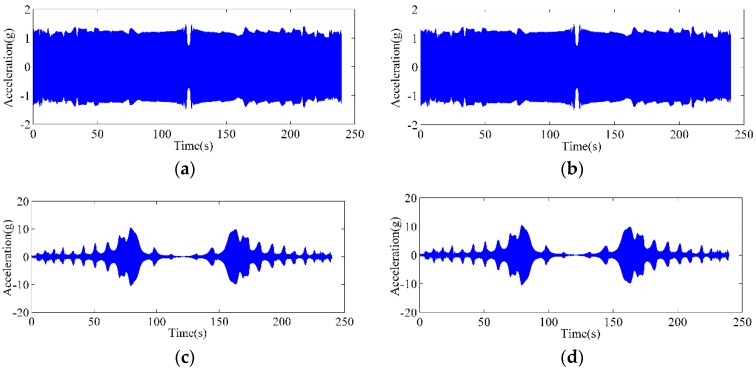
Time-domain waveforms of accelerations: (**a**) acceleration of the vibrator before any strands were broken; (**b**) acceleration of the vibrator after one strand was broken; (**c**) acceleration of the ACSR before any strands were broken; (**d**) acceleration of the ACSR after one strand was broken.

Figure 5 shows the time-domain waveform of the acceleration of the conductor and the shaker before and after a strand was broken. There is no clear difference in the acceleration signals in the diagram. Although the vibration amplitude of the shaker remains nearly unchanged, the acceleration amplitude of the conductor vibration is not constant; however, many peak points appear in the curves of the conductor, where resonance may occur. For a conductor, an elastic object whose mass is continuously distributed has multiple natural frequencies. Thus, the frequencies corresponding to the peak points in Figure 5a,b are the natural frequencies; however, the frequency values cannot be easily identified.

Typically, the frequency response function can intuitively reflect the modal parameters of a wire. The frequency response function is the self-power spectral density of excitation divided by the cross-spectral density of the wire and excitation, given by:

(6) $$H\left(\omega \right)=\frac{\ddot{x}(s)}{f(s)}\;$$

where *H*(*ω*) is the frequency response function, $\ddot{x}(s)$ is the conductor vibration acceleration after Fourier transformation, and f(s) is the vibrator exciting force after Fourier transformation. 

**Figure 6:**
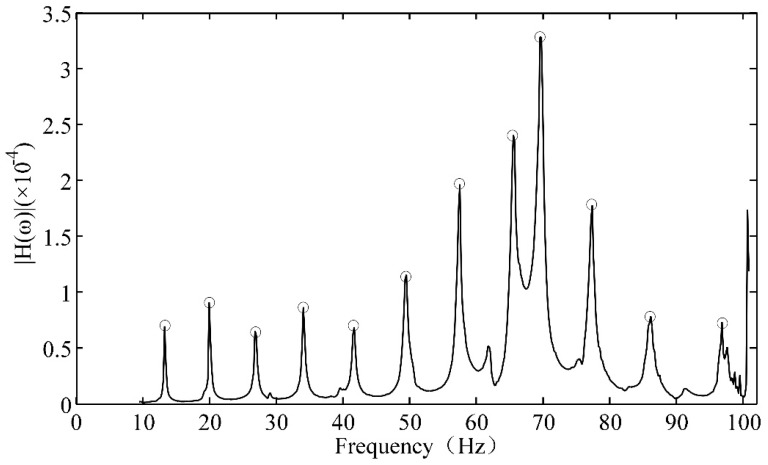
Frequency response function waveform of the intact wire.

**Figure 7:**
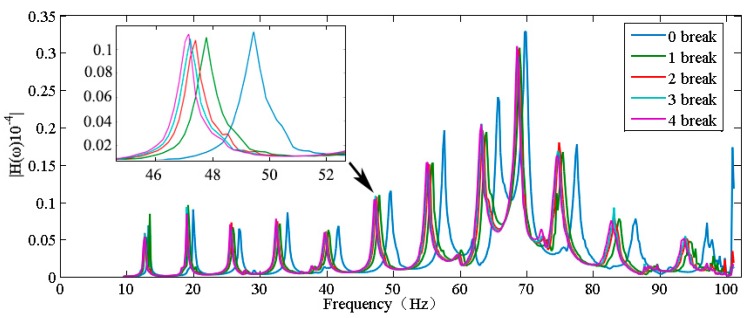
Frequency response function for a different number of broken strands.

Figure 6 shows the frequency response function waveform of the intact wire (i.e., no strands broken). The frequencies marked in the image are the natural frequencies. Figure 7 shows five frequency response function curves, each of which represents the frequency response function for a different number of broken strands. All the waveforms are similar, but the natural frequencies of all mode are different, and the modal frequency decreases considerably with an increasing number of broken strands.

To further verify the relationship between the number of broken strands and the natural frequency, an experiment was conducted with a maximum of four broken strands. When the number of broken strands was constant, the peak value of the frequency response function varied slightly, but the corresponding frequency values of the peak value were the same. Table 1 shows the values of the natural frequencies for a set of experiments. The modal frequency decreased with an increasing number of broken strands. Table 2 presents the absolute change (AC) and relative change (RC) in multiple natural frequencies with an increasing number of broken strands. The modal frequencies decreased significantly, especially when comparing intact strands with a single broken strand. The maximum absolute change was 2.67 Hz, and the maximum relative change was 3.9%. These results indicate that strand breakage can be effectively monitored by monitoring the change in natural frequency.

## 3. FBG-Based Monitoring System

**Figure 8:**
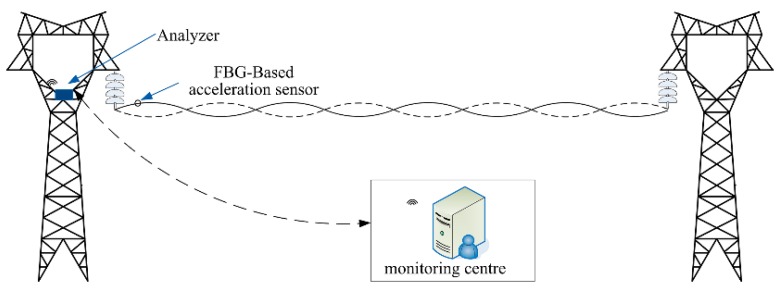
Overall diagram of the monitoring system. FBG = fiber Bragg grating.

A type of broken strand monitoring system for conductors is designed in this paper. The design is composed of an FBG-based accelerometer, analyzer, and monitoring center, as shown in Figure 8.

The FBG-based acceleration sensor is mounted on the conductor, which is designed to measure the acceleration of the conductor vibration. The FBG sensor model is SA-1201AF2D; the sensor measures 40 mm × 20 mm × 10 mm and has a measurement range of ±5 g. The mass of the sensor is less than 0.2 kg and thus has only a slight effect on the conductor. The acceleration sensor used in this paper contains a compensation grating fiber, which only measures temperature and does not measure acceleration. This compensation optical fiber is used to measure the wavelength change caused by temperature and considers this factor in Central Processing Unit (CPU) to reduce the error caused by temperature.

The wind speed sensor is installed on the tower to measure both wind speed and wind direction [17].

**Figure 9:**
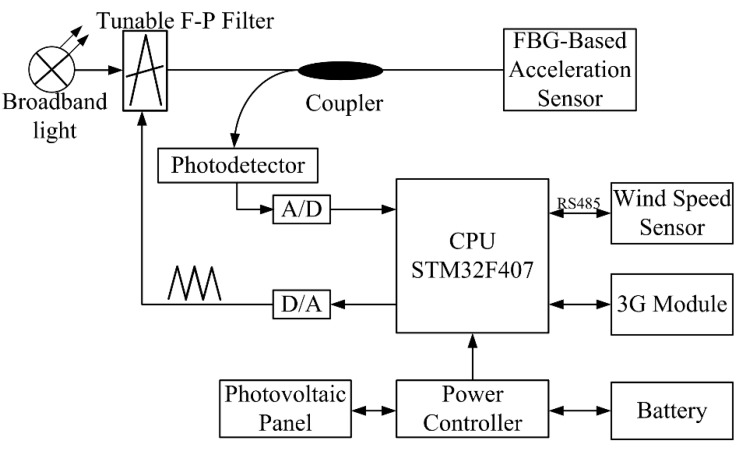
Block diagram of the analyzer.

The analyzer is installed on a tower to receive the data returned by the FBG and the wind speed sensor, and the natural frequency is calculated using those data; the data are then sent to the monitoring center via 3G. Because the data returned by the FBG are optical signals, the data must be converted into electrical signals before they can be processed. Figure 9 shows the hardware composition of the analyzer.

The light emitted by the broadband light source reaches the tunable Fabry-Perot (F-P) filter and the CPU output trigonometric wave through the digital-to-analog (DA) converter at the same time. Next, a tunable F-P filter carries out periodic scanning under a triangular wave voltage. The light wave that meets the peak transmission condition of the tunable F-P filter is transmitted, passes the coupler and reaches the FBG-based acceleration sensor. A beam of narrow band light, whose varied central wavelength reacts with the change in acceleration, reflects from the FBG. The reflected light is converted to an analog voltage signal by the photodetector (PD); then, the voltage signal is transformed into a digital signal via analog-to-digital (A/D) to the CPU. The STM32F407 chip, as the CPU of the analyzer, first receives both the acceleration signal and wind speed signal. Next, the chip calculates the natural frequency of the wire. Finally, the measured result is sent to the monitoring center through the 3G module. Two photovoltaic panels and a lead-acid battery are used in this system as the power supply.

## 4. Field Test and Discussion

### 4.1. Field Test

**Figure 10:**
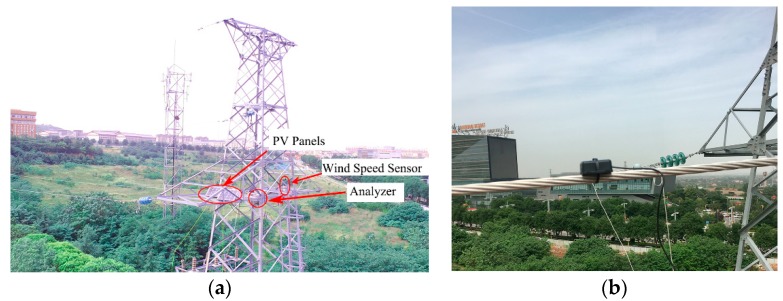
Field test of the FBG-based monitoring system. (**a**) Tower; (**b**) FBG-based acceleration sensor.

The performance of the FBG-based monitoring system was evaluated using a 105 m long transmission line span at Xi’an Polytechnic University. In this test, the voltage level of the transmission line was 110 kV, and the height of the conductor suspension point was 14 m. A wind speed sensor, the analyzer, and Photovoltaic (PV) panels were installed on the cross arm of the tower to measure wind speed, as shown in Figure 10a. The FBG-based acceleration sensor was mounted on one of the conductors, as shown in Figure 10b.

### 4.2. Discussion

**Figure 11:**
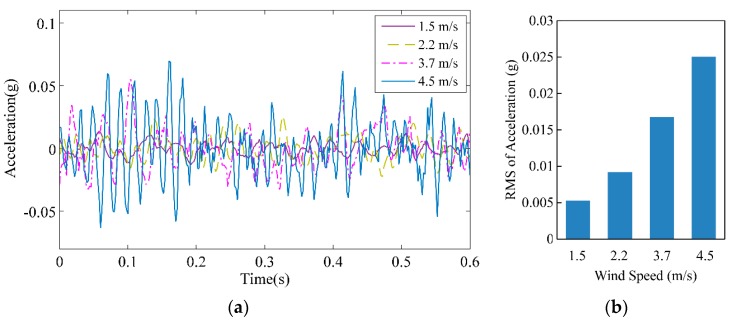
Acceleration responses of the conductor. (**a**) Vertical acceleration of the conductor; (**b**) root mean square (RMS) of the acceleration.

The field test recorded the response of conductor vibration on 23 May 2018 for wind speeds of 1.5, 2.2, 3.7, and 4.5 m/s, as shown in Figure 11a. The vibration was very weak when the wind speed was low, whereas the vibration became more distinct when the wind speed reached 4.5 m/s. Figure 11b shows the root mean square (RMS) of the acceleration. The vibration of the conductor increased with increasing wind speed. 

In the field test, no vibrator provided sweeping frequency excitation, and the excitation source was replaced by wind. In this case, modal parameter identification is usually called modal analysis under ambient excitation. Because of variations the wind speed, the dynamic response of the conductor is unstable. Therefore, time-varying analysis should be applied to analyze the vibration signals of the conductor. In this paper, STFT and SSI are used.

**Figure 12:**
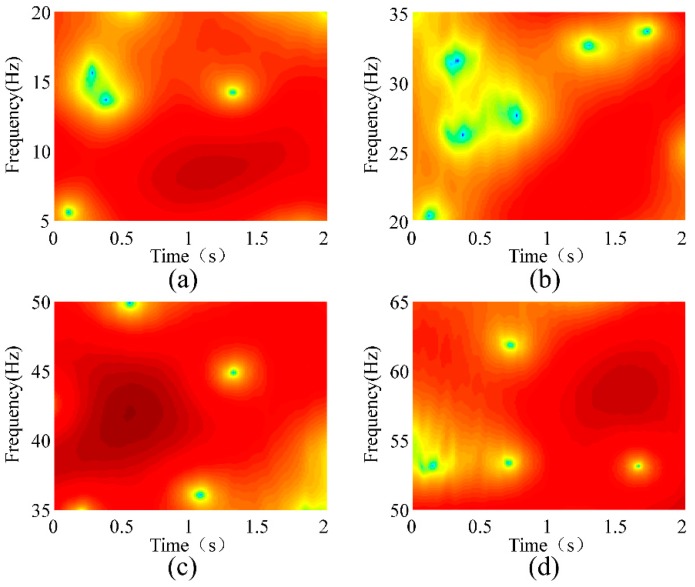
Short-time Fourier transform (STFT) analysis of the conductor’s vibration responses at a wind velocity of 1.5 m/s. (**a**) 1st Mode; (**b**) 2nd Mode; (**c**) 3rd Mode; (**d**) 4th Mode.

**Figure 13:**
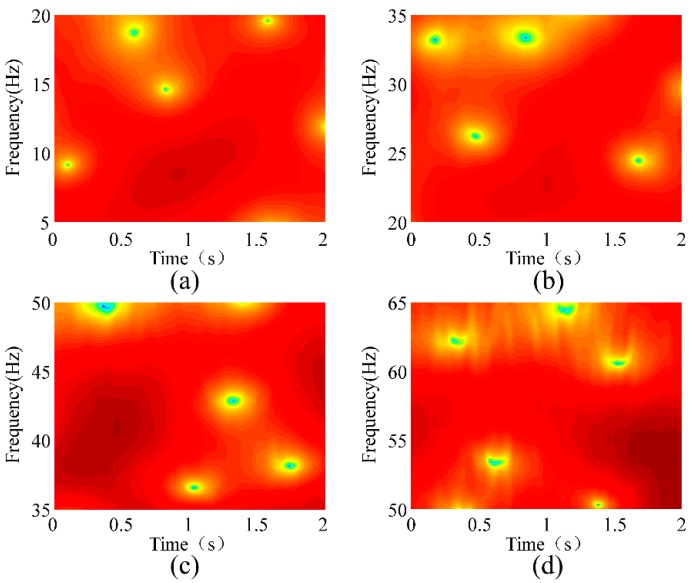
STFT analysis of the conductor’s vibration responses at a wind velocity of 2.2 m/s. (**a**) 1st Mode; (**b**) 2nd Mode; (**c**) 3rd Mode; (**d**) 4th Mode.

**Figure 14:**
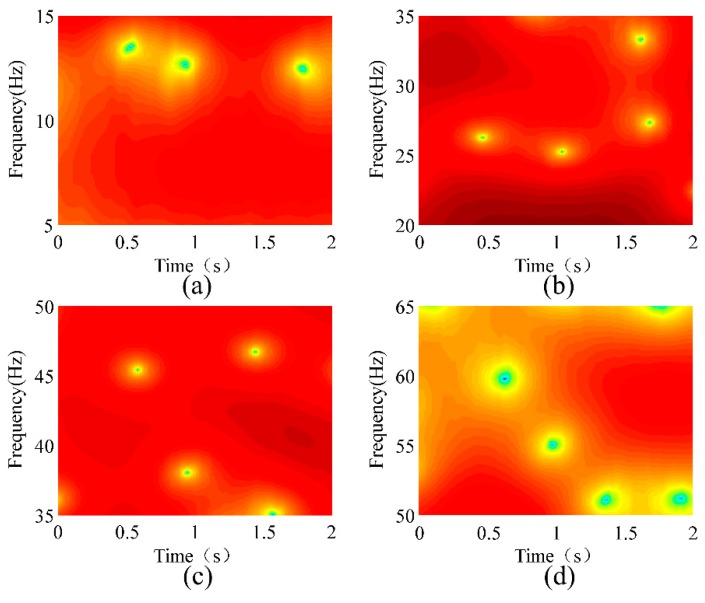
STFT analysis of the conductor’s vibration responses at a wind velocity of 3.7 m/s. (**a**) 1st Mode; (**b**) 2nd Mode; (**c**) 3rd Mode; (**d**) 4th Mode.

**Figure 15:**
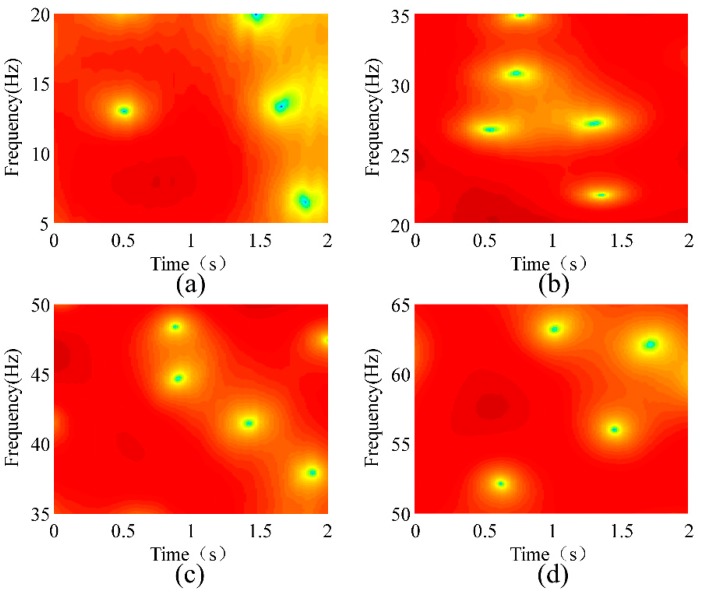
STFT analysis of the conductor’s vibration responses at a wind velocity of 4.5 m/s. (**a**) 1st Mode; (**b**) 2nd Mode; (**c**) 3rd Mode; (**d**) 4th Mode.

For the known time series of wire vibration, the STFT at time n can be expressed as:

(7) $$A\left(n,\omega \right)=\sum_{m=-\infty }^{\infty }a\left[m\right]\cdot w\left[n-m\right]\cdot e^{-j\omega m}\;$$

where *ω* is the frequency and *w*[*n*] is the window function. A rectangular window with a length of 128 was used for the STFT. Figure 12 shows the STFT analyses of the conductor’s vibration responses at a wind velocity of 1.5 m/s. Figure 12a–d show the natural frequencies of the 1st, 2nd, 3rd, and 4th modes, respectively. Figure 13, Figure 14 and Figure 15 show the STFT analyses under wind speeds of 2.2, 3.7, and 4.5 m/s, respectively. In these figures, the abscissa represents time, the ordinate represents frequency, and the depth of color indicates the intensity of vibration. 

The deepest part in the figure represents the strongest vibration, and the corresponding frequency is natural frequency. Table 3 lists the natural frequencies obtained using the STFT method. As the wind speed changes, the absolute error of the natural frequency obtained by the STFT reaches as high as 3.33 Hz. Compared with the conclusion of the second section, the error of this method is excessively high and thus is not suitable for detecting broken strands.

**Figure 16:**
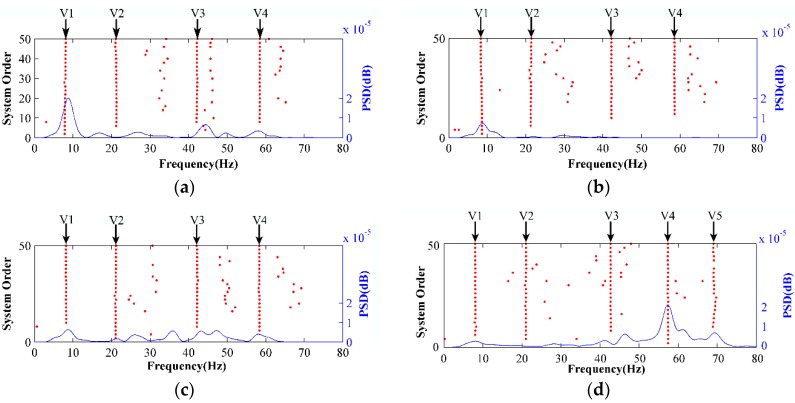
Stability chart and power spectrum density function (PSD) response of stochastic subspace identification (SSI) for modal identification of the conductor at wind velocities of (**a**) 1.5 m/s; (**b**) 2.2 m/s; (**c**) 3.7 m/s; (**d**) 4.5 m/s.

The SSI method was also used to analyze the vibration modes of four wind speeds. Figure 16 shows the stability chart analysis under four wind speeds. When the wind speed is 1.5, 2.2, and 3.7 m/s, even if the power spectrum density function (PSD) was not prominent, the natural frequencies of four vertical modes were extracted. At wind speed of 4.5 m/s, the fifth vertical mode could be extracted.

Table 4 shows the natural frequencies calculated using the SSI method. The absolute error is considerably smaller than that obtained using the STFT method. Compared with the conclusion of the second section, the maximum absolute error of this method is considerably smaller than the absolute variation of frequencies caused by the broken strands. Therefore, significant changes in the natural frequency calculated by the SSI method indicate that the strand structure is broken and requires on-site maintenance.

As can be seen in the tables, the maximum absolute errors of the 3rd and 4th modes of the SSI method under the four wind speeds are 0.42 and 0.38 respectively, and they are much smaller than those of STFT. To further illustrate the accuracy of the two methods, more experimental data are analyzed in this paper. We used 10 sets of measured data for statistical analysis, with wind speeds ranging from 1.3 m/s to 5 m/s. The analysis results are shown in Table 5. The Standard Deviation (SD) of STFT method is much higher than that of SSI method.

## 5. Conclusions

In this paper, a new method for detecting broken strands of a transmission line conductor using modal parameter identification was proposed based on decreases in stiffness and natural frequency after strands break. The theory was verified by a frequency sweep test of a vibrator. The experimental results showed that the natural frequencies of the transmission lines will decrease after a strand is broken, and the natural frequency will decline further with increases in the number of broken strands. Based on the results, the maximum absolute change was 2.67 Hz, and the maximum relative change was 3.9%. Therefore, this method is feasible.

According to the feasibility experiment of this method, an FBG-based monitoring system was designed considering the special electromagnetic environment of the transmission line. The system has the advantages of anti-electromagnetic interference and light weight. The system was implemented on a 105 m long transmission line span at Xi’an Polytechnic University, with four types of vibration signals under different wind speeds collected and the modal parameters identified. The STFT method and SSI method were used to analyze the measured data. The results showed that the maximum absolute errors of the 3rd and 4th modes of the SSI method under the four wind speeds were 0.42 and 0.38, respectively. In addition, the absolute errors were considerably smaller than those obtained using the STFT method; thus, the SSI method can be used to extract natural frequencies in the field.

## Figures and Tables

**Table 1:** Change in natural frequency with the number of broken strands.

	0 Strand	1 Strand	2 Strands	3 Strands	4 Strands
1st mode	13.206	12.825	12.634	12.629	12.625
2nd mode	19.882	19.119	19.024	18.929	18.916
3rd mode	26.844	25.890	25.699	25.604	25.509
4th mode	34.092	32.757	32.471	32.375	32.280
5th mode	41.721	40.196	39.814	39.718	39.623
6th mode	49.446	47.824	47.443	47.253	47.062
7th mode	57.552	55.740	55.168	55.072	54.977
8th mode	65.659	63.656	63.274	63.083	62.988
9th mode	69.569	68.710	68.614	68.424	68.424
10th mode	77.293	75.291	74.719	74.432	74.337
11th mode	86.067	83.587	82.824	82.816	82.538
12th mode	96.844	94.173	93.601	93.595	93.315

**Table 2:** Change in natural frequency with the number of broken strands.

	0 to 1 Strand		1 to 2 Strands		2 to 3 Strands		3 to 4 Strands	
	AC ^1^	RC	AC	RC	AC	RC	AC	RC
1st mode	0.381	2.89%	0.191	1.49%	0.005	0.04%	0.004	0.03%
2nd mode	0.763	3.84%	0.095	0.50%	0.095	0.50%	0.013	0.07%
3rd mode	0.954	3.55%	0.191	0.74%	0.095	0.37%	0.095	0.37%
4th mode	1.335	3.92%	0.286	0.87%	0.096	0.30%	0.095	0.29%
5th mode	1.525	3.66%	0.382	0.95%	0.096	0.24%	0.095	0.24%
6th mode	1.622	3.28%	0.381	0.80%	0.190	0.40%	0.191	0.40%
7th mode	1.812	3.15%	0.572	1.03%	0.096	0.17%	0.095	0.17%
8th mode	2.003	3.05%	0.382	0.60%	0.191	0.30%	0.095	0.15%
9th mode	0.859	1.23%	0.096	0.14%	0.190	0.28%	0.000	0.00%
10th mode	2.002	2.59%	0.572	0.76%	0.287	0.38%	0.095	0.13%
11th mode	2.480	2.88%	0.763	0.91%	0.008	0.01%	0.278	0.34%
12th mode	2.671	2.76%	0.572	0.61%	0.006	0.01%	0.280	0.30%

^1^ AC stands for absolute change and RC stands for relative change.

**Table 3:** Natural frequencies calculated by short-time Fourier transform (STFT).

Wind Speed (m/s)	Mode V1 (Hz)	Mode V2 (Hz)	Mode V3 (Hz)	Mode V4 (Hz)
1.5	8.575	23.05	42.18	58.69
2.2	8.453	22.92	41.11	55.36
3.7	8.026	20.51	40.68	58.14
4.5	8.026	20.63	40.25	57.83
Maximum absolute error	0.549	2.54	1.93	3.33

**Table 4:** Natural frequencies calculated by stochastic subspace identification (SSI).

Wind Speed (m/s)	Mode V1 (Hz)	Mode V2 (Hz)	Mode V3 (Hz)	Mode V4 (Hz)	Mode V5 (Hz)
1.5	8.131	21.9	42.09	58.42	-
2.2	8.08	21.87	41.90	58.6	-
3.7	8.146	21.69	42.04	58.54	-
4.5	8.08	21.56	42.32	58.22	69.53
Maximum absolute error	0.066	0.34	0.42	0.38	-

**Table 5:** Statistical analysis results.

	STFT				SSI			
	Mode V1	Mode V2	Mode V3	Mode V4	Mode V1	Mode V2	Mode V3	Mode V4
Maximum	8.575	23.05	42.63	58.93	8.228	22.07	42.32	58.69
Minimum	7.965	20.51	40.25	55.36	7.923	21.56	41.69	58.22
RSD ^1^	8.242	21.864	41.656	57.456	8.083	21.873	41.977	58.443
SD	0.184	0.985	0.754	1.242	0.079	0.156	0.201	0.151

^1^ RSD is the abbreviation of “relative standard deviation”.
